# Compliance with clinical pathways for inpatient care in Chinese public hospitals

**DOI:** 10.1186/s12913-015-1121-8

**Published:** 2015-10-06

**Authors:** Xiao Yan He, M. Kate Bundorf, Jian Jun Gu, Ping Zhou, Di Xue

**Affiliations:** Department of Hospital Management, Key Laboratory of Health Technology Assessment (MOH), Collaborative Innovation Center of Social Risks Governance in Health, School of Public Health, Fudan University, No 138, Yi Xue Yuan Road, P.O.Box 197, Shanghai, 200032 P. R. China; School of Medicine, Stanford University, HRP Redwood Building, 259 Campus Drive, Stanford, CA 94305-5405 USA; Commission of Health and Family Planning of Pudong New Area, No. 990, Chengshan Road, Shanghai, 200125 P. R. China

**Keywords:** Clinical pathway, Compliance, Chart audit, Hospital

## Abstract

**Background:**

The National Health and Family Planning Commission of China has issued more than 400 clinical pathways to improve the effectiveness and efficiency of medical care delivered by public hospitals in China. The aim of our study is to determine whether patient care is compliant with national clinical pathways in public general hospitals of Pudong New Area in Shanghai.

**Methods:**

We identified the clinical pathways established by the National Health and Family Planning Commission of China for 5 common conditions (community-acquired pneumonia, acute myocardial infarction (AMI), heart failure, cesarean section, type-2 diabetes). We randomly selected patients with each condition admitted to one of 7 public general hospitals in Pudong New Area in China in January, 2013. We identified key process indicators (KPIs) for each pathway and, based on chart review for each patient, determined whether the patient’s care was compliant for each indicator. We calculated the proportion of care which was compliant with clinical pathways for each indicator, the average proportion of indicators that were met for each patient, and the proportion of patients whose care was compliant for all measures. For selected indicators, we compared compliance rates among hospitals in our study with those from other countries.

**Results:**

Average compliance rates across the KPIs for each condition ranged from 61 % for AMI to 89 % for pneumonia. The percent of patient receiving fully compliant care ranged from 0 for AMI and heart failure to 39 % for pneumonia. Compared to the compliance rate for process indicators in the hospitals of other countries, some rates in the hospitals that we audited were higher, but some were lower.

**Conclusions:**

Few patients received care that complied with all the pathways for each condition. The reasons for low compliance with national clinical pathways and how to improve clinical quality in public hospitals of China need to be further explored.

**Electronic supplementary material:**

The online version of this article (doi:10.1186/s12913-015-1121-8) contains supplementary material, which is available to authorized users.

## Background

Ensuring that hospitals consistently provide high quality care is a challenge facing policymakers and hospital administrators around the world. The quality of hospital care is an important issue for policy makers in China as patients are increasingly demanding higher quality care. A key component of national policies intended to improve quality of care has been the development and use of clinical pathways.

As in many other countries, the use of clinical pathways has increased rapidly in China in recent years. The National Health and Family Planning Commission (NHFPC, previously called “Ministry of Health”) of China has issued more than 400 clinical pathways [1]. Despite the emphasis placed on the use of pathways, there is little evidence on the extent to which Chinese hospitals provide care consistent with these pathways [2].

### Clinical guidelines and pathways

Clinical guidelines are recommendations on the appropriate treatment and care of people with specific diseases and conditions [3]. Clinical pathways, in contrast, support the translation of clinical guidelines into local practice by identifying the specific steps necessary to translate the clinical guideline into practice in a particular local environment [4]. By linking evidence to clinical practice, the use of clinical pathways is intended to optimize patient outcomes and increase clinical efficiency [4].

In China, national medical associations generally create clinical guidelines and the NHFPC translates the guidelines into clinical pathways. In 2012, the NHFPC required every tertiary- and secondary-level hospital in China to implement at least 60 clinical pathways, with at least 40 from among the over 400 established by the NHFPC, although the hospitals may customize the pathways for their patients. In this study, we examine the extent to which the care provided by public hospitals in Shanghai is consistent with national clinical pathways.

### Effects of clinical guidelines and pathways

Studies have documented an association between the use of clinical guidelines and pathways and positive outcomes including the provision of high-quality, cost-effective care, greater patient and staff satisfaction, and better resource management in a variety of clinical contexts [4–7]. Other studies have documented that the adoption of clinical pathways can reduce length of stay and decrease medical cost [8–10].

In this study, we document the clinical pathways established by the NHFCP for five clinical conditions: community-acquired pneumonia (“pneumonia”), AMI, heart failure, cesarean section, type-2 diabetes. Clinical guidelines or pathways have been shown to be effective in these clinical contexts. Guideline-concordant therapy for community-acquired pneumonia is associated with improved health outcomes and the use of fewer resources [11, 12]. Compliance with acute myocardial infarction (AMI) guidelines is associated with lower inpatient mortality [13–15] and the implementation of a clinical pathway for heart failure was associated with improvements in care processes as well as reduced length of stay and hospital charges [16, 17]. A study of the implementation of National Institute for Health and Clinical Excellence (NICE) guidance regarding caesarean section documented lower rates of surgical site infection following caesarean section [18]. In the context of diabetes, the implementation of a process improvement effort using practice guidelines resulted in greater compliance with recommended HbA1c, lipid, blood pressure, and foot checks, leading to better control of blood pressure and lower body mass index (BMI) [19]. Despite the potential for adherence to clinical guidelines to reduce mortality and morbidity and decrease healthcare costs, there is substantial evidence that adherence to guidelines in clinical practice is often poor [13, 14, 20–22].

In our analysis, we measure the extent to which the care patients received was compliant with the national clinical pathways for these conditions in public general hospitals of Pudong New Area in Shanghai. We identify key process indicators (KPIs) for each condition based on the clinical pathways and then determine whether the care of randomly selected patients with each condition was consistent with these indicators. We also compare performance on several clinical pathways with results from studies of other countries.

Our study is the first to document the extent to which public hospitals in China are adhering to national guidelines. The study provides important baseline information on the delivery of health care in Chinese public hospitals and the potential for improvements in health care quality.

## Methods

### Survey sample

We studied physician compliance with the national clinical pathways in all seven public, general hospitals in Pudong New Area of Shanghai. We chose to study 5 conditions: pneumonia, AMI, heart failure, cesarean section, and type-2 diabetes. These conditions were among the top ten in patient volume in all the surveyed hospitals and had national clinical pathways published by NHFPC [23].

Using hospital information systems, we identified all patients with a given diagnosis, based on inpatient international classification of diseases (ICD-10 or ICD-9) codes, admitted to each hospital during 2012 for each condition. To ensure that the sample was evenly distributed throughout the year, we randomly selected the first two inpatient admissions with an odd patient number for each condition in each month. If a hospital admitted fewer than 24 patients for a particular condition in 2012, then all the medical records for this condition were extracted for this hospital.

### Data sources

We developed an audit chart for each of the five conditions based on the clinical pathways published by NHFPC. The audit chart identified the key process components in the clinical pathway, focusing on those both that were important determinants of quality of care and for which data was likely to be available in medical records. We then extracted data from the medical records corresponding to each item in the audit chart for each patient.

To ensure the quality and consistency of chart audit, we trained five researchers on the meanings of each item on the checklist and how to audit each chart. The researchers then observed two experts auditing charts to assess compliance for heart failure and cesarean section pathways in one hospital and subsequently audited the same charts the experts audited. The consistency between the experts and the researchers for these two conditions was 87 %.

For each admission, we also collected data on patient demographics and health status as well as some financial information from the hospital information systems (HIS) of the surveyed hospitals.

### Selection of key process indicators

The national clinical pathways are very detailed and when we abstracted data, we tried to gather information on each step. When reporting the results, we chose to focus on the more clinically meaningful components of each pathway (see Additional file 1). For example, in the pneumonia pathway, we focused on severity assessment and corresponding treatment, appropriate use of antibiotics, health education, and appropriate length of stay. We did not include appropriateness of admission as a KPI. Similarly, for AMI, we focused on timely treatment and evaluation of left ventricular function, appropriate use of medicine (such as aspirin/clopidogrel, β-blocker, ACEI/ARB, statins), reperfusion therapy, thrombolytic therapy and health education, because they are life-saving and important for secondary disease preventions. We did not include length of stay for AMI due to the potential for differences across patients in appropriate length of stay.

### Data analysis

We coded hospitals as compliant for an indicator only if the information was recorded in the medical record and the care was consistent with the clinical pathway or if the medical record included a reasonable explanation for not being compliant. For each KPI, we calculated the proportion of patients who received compliant care.

From this information, we also calculated two patient-level measures of compliance: 1) whether the patient received pathway compliant care for all indicators (fully compliant care) and 2) the proportion of KPIs that were met for the patient. We used these patient-level measures to calculate the proportion of patients receiving fully compliant care (full compliance rate) for each condition and the average of the proportion of KPIs that were met over all patients with a given condition (average compliance rate).

### Ethics approval

This study was approved by Institutional Review Board, School of Public Health, Fudan University (IRB#2012-11-0383).

### Consent statement

N/A for this retrospective study.

## Results

The numbers of medical records audited across all hospitals in the study were 151, 97, 145, 146 and 137 for pneumonia, AMI, heart failure, caesarean section, and type-2 diabetes, respectively (Tables 1, 2, 3, 4 and 5).

**Table 1:** Compliance rates for KPIs for inpatient care of pneumonia (*n* = 151)^a^

No	Key process indicators	No of Cases	Compliance rate (%)
1	Patient severity assessed	151	95
2	Severe patients (oxygen saturation <92 %) received blood gas analysis	151	70
3	Timeliness of sputum and blood culture	151	77
4	Timely and appropriate use of antibiotics within 4 h	150	92
5	Appropriate treatment update at 72 h	151	98
6	Antibiotic treatment is reasonable (7 ~ 14 days)	151	81
7	Received health education	151	98
8	Appropriate length of stay	151	100
	Average		89

^a^ *KPIs* key process indicators

**Table 2:** Compliance rates for KPIs for inpatient care of AMI (*n* = 97)^a^

No	Key process indicators	No of Cases	Compliance rate (%)
1	Timely use of aspirin or clopidogrel in appropriate dosage	95	68
2	Evaluation of left ventricular function within 24 h of admission	96	84
3	Reassessment of patient condition within one week before discharge	96	0
4	Reperfusion therapy	89	75
5	Thrombolytic therapy within 30 min of admission	97	5
6	PCI within 90 min of admission	97	0
7	Use of β-blocker within 60 min of admission	96	25
8	Use of aspirin during hospitalization	96	94
9	Use of β-blocker during hospitalization	97	73
10	USE of ACEI or ARB during hospitalization^b^	97	73
11	Use of statins during hospitalization	97	93
12	Cholesterol test and lipid lowering therapy	97	27
13	Advised to continue to use aspirin after discharge	97	80
14	Advised to continue to use β-blocker after discharge	96	61
15	Advised to continue to use ACEI or ARB after discharge	94	65
16	Advised to continue to use statin after discharge	97	78
17	Advised to no smoking, having exercise, healthy eating, weight control, proper treatment of reoccurrence or worsening, etc.	97	85
18	Smoking cessation counseling	97	85
19	Provided with written instruction on secondary prevention in discharge summary	97	84
	Average		61

^a^ *KPIs* key process indicators

^b^ *ACEI* angiotensin converting enzyme inhibitors,ARB:Angiotensin II receptor blockers

**Table 3:** Compliance rates for KPIs for inpatient care of heart failure (*n* = 145)^a^

No	Key process indicators	No of Cases	Compliance rate (%)
1	Assessment of left ventricular function within 24 h of admission	133	77
2	Assessment of left ventricular function 1 week prior to discharge	121	1
3	Timely use of diuretics and potassium agents	140	96
4	Timely use of ACEI or ARB^b^	145	87
5	Use of β-blockers only for patients with CHF^c^	133	20
6	Use of aldosterone receptor blockers only for patients with severe health failure^d^	142	85
7	Continued use of diuretics during hospitalization	145	93
8	Continued use of ACEI or ARB during hospitalization	144	87
9	Continued use of β-blocker during hospitalization	129	53
10	Continued use of aldosterone receptor blockers during hospitalization	139	84
11	Advised to use diuretics after discharge	139	82
12	Advised to use ACEI or ARB after discharge	140	76
13	Advised to use β-blocker after discharge	126	54
14	Advised to use aldosterone receptor blockers after discharge	134	78
15	Record of heart failure education	145	100
16	Assessment of cardiac function and living ability, and guidance activities after admission	145	100
17	Proper observation of patients (including symptoms, vital signs, water balance, weight, edema), provision of laboratory tests, and advice on diet and body-position after admission.	144	100
18	Assessment of tobacco and alcohol addiction after admission and Patient advised to quit smoking and to restrict alcohol consumption	145	54
19	Patient received psychological counseling	145	59
20	Patient advised on activity limitations after discharge	144	100
21	Patient received dietary and body-position guidance prior to discharge	143	100
22	Patient advised to quit smoking and to restrict alcohol consumption prior to discharge	143	99
	Average		78

^a^ *KPIs* key process indicators

^b^ *ACEI* angiotensin converting enzyme inhibitors, *ARB* angiotensin II receptor blockers

^c^ *CHF* chronic heart failure

^d^Severe heart failure refers to the New York Heart Association (NYHA) cardiac function proposed test (NYHA functional) III, IV level of the patients

**Table 4:** Compliance rates of KPIs for inpatient caesarean section (*n* = 146)^a^

No	Key process indicators	No of Cases	Compliance rate (%)
1	Appropriate indication for planned C-section	146	100
2	Preoperative examination completed within 2 days	146	99
3	Prophylactic use of first generation cephalosporin antibiotics	146	62
4	Withdraw of prophylactic antibiotics within 72 h after delivery	146	86
5	The timeliness of operation time	146	62
6	Delivery within 2 days of admission	146	62
7	Appropriate anesthesia	145	66
8	Appropriate use of oxytocin during procedure	146	81
9	Post-operative length of stay	143	99
10	In accordance with discharge standard	142	100
11	Patient received health education prior to discharge	144	89
	Average		82

^a^ *KPIs* key process indicators

**Table 5:** Compliance rates of KPIs for inpatient care of type-2 diabetes (*n* = 137)^a^

No	Key process indicators	No of Cases	Compliance rate (%)
1	Routine examination within 24 h after admission	137	100
2	Blood glucose monitoring 7 times per day	137	64
3	HbA1c test	137	91
4	Glycosylated Serum Protein (Fructosamine) test	136	55
5	OGTT and insulin or C peptide release test^b^	137	58
6	Eye fundus examination	137	62
7	Nerve system examination	137	39
8	Renal function examination	137	83
9	Heart ultrasound examination	137	72
10	Carotid artery and lower extremity vascular ultrasound examination	137	73
11	Blood glucose test analyzed	137	97
12	Evaluation at 72 h after hypoglycemic treatment	137	85
13	Record of drug selection reasons	137	99
14	Record of secondary prevention and health education provided to patient	137	98
15	In accordance with discharge standard	137	96
16	Appropriate length of stay	137	88
	Average		79

^a^ *KPIs* key process indicators

^b^ *OGTT* oral glucose tolerance test

### Compliance rates

Compliance rates for the KPIs for pneumonia ranged from 70 to 100 %. All the patients with pneumonia had appropriate length of stay according the pathway, but the compliance rate for “Severe patients (defined as oxygen saturation < 92 %) received blood gas analysis” was 70 %. The proportion of patients who received initial antibiotics properly within 4 h of hospital arrival in our study was 92 % (Table 1).

The compliance rates for the AMI KPIs ranged from 0 to 94 %. The lowest three compliance rates were for “Reassessment of patient condition within 1 week before discharge” (0 %), “PCI(percutaneous coronary intervention) within 90 min of admission” (0 %), and “Thrombolytic therapy within 30 min of admission” (5 %). In addition, the compliance rate for reperfusion therapy for STEMI(ST - segment elevation myocardial infarction) or LBBB(left bundle branch block) patients was 75 % and for using β-blocker within 24 h of admission was 67 %. Eighty percent, 61 and 65 % of AMI patients were advised to continue to use aspirin, β-blocker, and ACEI(angiotensin-converting enzyme inhibitor)/ARB (angiotensin receptor antagonist) after discharge, respectively. Forty-eight percent of inpatients without a contraindication of heart failure did not receive β-blockers (Table 2).

The compliance rates for the heart failure KPIs varied widely, ranging from 1 to 100 %. Rates were lowest for “Assessment of left ventricular function 1 week prior to discharge” (1 %) and for “Use of β-blockers only for patients with chronic heart failure” (20 %). In addition, the proportion of inpatients not using β-blockers without a documented reason was 48 % (Table 3).

The compliance rates for the caesarean section KPIs ranged from 62 to 100 %. The three KPIs with the lowest compliance rates were “The timeliness of operation time” (62 %), “Prophylactic use of first generation cephalosporin antibiotics” (62 %), and “Delivery within 2 days of admission” (62 %). The compliance rate for appropriate use of oxytocin (10ug or 20ug) during cesarean section was quite high (81 %) (Table 4).

The compliance rates for the type-2 diabetes KPIs ranged from 39 to 100 %. The three KPIs with the lowest compliance rates were “Nerve system examination” (39 %), “Glycosylated serum protein (Fructosamine) test” (55 %), and “Oral glucose tolerance test (OGTT) and insulin or C peptide release test” (58 %). The compliance rate for HbA1c test was 91 % (Table 5).

The compliance rates for health education for all five diseases were relatively high (above 86 %) in the surveyed hospitals (Tables 1-5).

### Full and average compliance rates

The proportion of patients who received fully compliant care was low for each of the five conditions in the surveyed hospitals, ranging from 0 % (AMI and heart failure) to 39 % (pneumonia). The average compliance rates of the KPIs ranged from 61 % (AMI) to 89 % (pneumonia). The compliance rates among the 5 selected conditions were significantly different (Table 6).

**Table 6:** Analysis on compliance rate for inpatient care

		Full compliance rate			Average compliance rate		
Indicators	No of KPIs^a^	Number of cases	Number of fully compliant cases	Percent (%)	Average number of cases for KPVs	Average cases that meet the requirement for KPVs	Percent (%)
Pneumonia	8	150	58	39	151	134	89
AMI	19	80	0	0	96	59	61
Heart failure	22	76	0	0	139	108	78
Caesarean	11	139	32	23	145	119	82
Type-2 diabetes	16	136	3	2	137	108	79
Total	76	581	93	16	668	528	79
*χ* ^2^			111.37***			27.45***	
P_fisher’s exact test_			1.560E-26			2.783E-05	

***P < 0.01

^a^KPIs Key Process Indicators

## Discussion

### Compliance rates for the five conditions

The objectives of the use of clinical pathways are to improve quality of care, to reduce costs, and to decrease inappropriate variation in health care use [24, 25]. Our analysis shows, however, that the establishment of extensive pathways for Chinese hospitals has not led to highly compliant care. The proportion of patients receiving fully compliant care ranged from 0 % (for AMI and heart failure) to 39 % (for pneumonia). Average compliance rates across all indicators for patients with a given condition ranged from 61 % for AMI to 89 % for pneumonia.

In our study, we considered a hospital non-compliant for a particular indicator if the information was not available in the medical record. Thus while the lack of compliance for the KPIs we examined could be driven by non-compliant care, it could also be due to a lack of documentation. It is possible that the care patients receive may be more compliant with clinical pathways than our results suggest, and that hospitals could potentially improve their measured performance through better documentation.

It is also possible that our findings are influenced by the timing of the study. The national pathways were issued relatively recently in 2009 and the importance of adhering to pathways may not have been fully valued by hospital managers in 2012. A qualitative study of the use of clinical pathways in Chinese hospitals identified lack of leadership and support for implementing clinical pathways as barriers to compliance [26].

Low rates of compliance with clinical pathways could also reflect physician concerns over the quality of the pathway. The quality of clinical pathways is dependent upon the quality of the underlying evidence, and, for many clinical applications, the evidence base is inadequate. Even in situations with adequate evidence, clinical pathways could adversely affect patient care if the evidence is not translated effectively into the clinical pathways. In addition, simplistic clinical pathways may not accommodate heterogeneity of patients in practice. An alternative explanation for low rates of compliance is that, in some cases, physicians may believe that guideline compliant care is inappropriate for patients.

We note that we evaluated whether care was consistent with national pathways but did not evaluate whether the national pathways were appropriate. Similarly, hospitals were able to customize the national pathways for their local setting, providing another potential explanation for the deviations from the national pathways that we observed [22]. Determining the extent to which the pathways represent appropriate care is important for evaluating the desirability of increasing rates of compliance with national pathways.

Clinical pathways may also be difficult to implement. Physicians may be concerned that using clinical pathways will reduce their autonomy. Other barriers include a lack of incentives to change practice styles, unclear accountability for health outcomes, competing priorities, lack of resources, health funding constraints, regulation and patient factors (clinical contraindications or history of intolerance to a recommended medication, patient refusal), difficulty coordinating across providers and cultural barriers [6, 26–30]. Correspondingly, studies generally document relatively low levels of compliance with clinical pathways although compliance level varies substantially across sites and across measures.

In our study, differences across conditions in the degree of compliance may have been driven by condition complexity. For example, in the case of pneumonia, treatment does not vary much across patients. In the case of AMI, in contrast, not only is treatment urgent, but guideline compliant care varies significantly across patients. Similarly, heart failure is more complex than cesarean section. Consistent with this explanation, we found the highest rates of non-compliance among AMI and heart failure patients, the two most complex conditions we studied.

### Comparison of compliance rates with other countries

The compliance rates for process indicators in public general hospitals of Pudong new area were higher than those from studies from other countries in some cases and were similar or lower in others. For example, 92 % of patients with pneumonia in our study received initial antibiotics within 4 h of hospital arrival, compared to 81 % within 8 h in a study in the U.S. [20]. According to our chart review, 91 % of type-2 diabetics received an HbA1c test, but this rate was about 32 % in the hospitals in the eastern province of Saudi Arabia [31].

But, for patients with AMI, only 5 % received thrombolytic therapy within 30 min of admission, 0 % received PCI within 90 min of admission, and 61 % were advised to continue to use β-blocker after discharge, much lower than corresponding rates in U.S. studies (36, 70 and 79 %, respectively) [13]. The proportion of inpatients with heart failure inappropriately not using β-blockers in our study was 48 %, much higher than that in the U.S. (15 %) [27].

The compliance rates for using β-blocker within 24 h, for advising to continue to use aspirin after discharge, and for advising to continue to use ACEI/ARB after discharge for AMI in our study were 67, 80, and 65 %, similar to those in US and French studies (59.5–74 %, 72–87 %, 53–80 %) [13–15, 32, 33].

The compliance rate for appropriate use of oxytocin during cesarean section was quite high (81 %). In contrast, a survey of 306 clinicians on the implementation of a pathway on oxytocin use during cesarean section in England and Wales found that only 40 % of the surveyed clinicians followed the NICE recommendation [34]. While these comparisons should be interpreted with caution since they are based on studies of different time periods and clinical practice changes rapidly, they point to important differences across countries in the extent to which health care organizations provide pathway compliant care.

### Limitations

The findings of this study were based on chart review of 5 common conditions in inpatient care of 7 public general hospitals of Pudong new area in Shanghai. As a result, the study results may not represent the experience of other areas or regions of China, although they are likely to reflect medical practice in Shanghai. We emphasize, however, that Pudong New Area includes both urban and rural areas, indicating that our study sample includes economically diverse patients. In general, because the socioeconomic status is higher and more resources are devoted to health care in eastern China than in other areas, the study results are likely to represent an area in China with relatively high quality of care.

## Conclusions

Evidence-based clinical pathways may be used to improve quality of care, reduce costs, and decrease inappropriate variation in clinical practice. But our study found that public general hospitals in Shanghai had low compliance with national clinical pathways for 5 common conditions (pneumonia, AMI, heart failure, cesarean section, type-2 diabetes). The results, which provide unique evidence of the state of quality of care in Chinese public hospitals, indicate that opportunities exist to improve quality of care in Chinese public hospitals.

Our study also suggests that Chinese public hospitals could use the clinical pathways established by the NHFPC as a tool to identify areas in which quality could be improved. Our audit study identified certain KPIs for which Chinese hospitals performed well and others for which they did not. This information could be used to help managers in Chinese public hospitals direct quality improvement activities.

Finally, our results suggest that opportunities exist for the NHFPC to create and for hospitals to use clinical pathways more effectively. Our findings indicated that hospitals in Shanghai often do not provide care consistent with clinical pathways. More research is necessary to determine if the pathways are appropriate and, if so, how to encourage hospitals to change processes of care to be consistent with the guidelines.

## Additional file

Additional file 1:
**Analyses on compliance rates for 5 conditions.** (DOCX 47 kb)

## Abbreviations

NHFPC: National Health and Family Planning Commission
AMI: Acute myocardial infarction
NICE: National Institute for Health and Clinical Excellence
BMI: Body mass index
HIS: Hospital information systems
KPIs: Key process indicators
PCI: Percutaneous coronary intervention
STEMI: ST - segment elevation myocardial infarction
LBBB: Left bundle branch block
ACEI: Angiotensin-converting enzyme inhibitor
ARB: Angiotensin receptor antagonist
OGTT: Oral glucose tolerance test
