# Supplementary material for: Compliance with clinical pathways for inpatient care in Chinese public hospitals
Source: BMC Health Serv Res. 2015 Oct 6;15:459. doi: 10.1186/s12913-015-1121-8 (PMC4595105; doi:10.1186/s12913-015-1121-8)
Supplement: Additional file 1: — Analyses on compliance rates for 5 conditions. (DOCX 47 kb) [file 12913_2015_1121_MOESM1_ESM.docx]

Additional file 1: Analyses on compliance rates for 5 conditions

Table S1 Analysis on Compliance Rate for Pneumonia Inpatient Care (n=151)^†^

| Key process indicators | | | N | Percentage（%） | Rule | Missing Data | Compliance rate (%) |
| --- | --- | --- | --- | --- | --- | --- | --- |
| Patient severity assessed | | |  |  | A | 0 | 94.70 |
|  | A | yes | 143 | 94.70 |  |  |  |
|  | B | no | 8 | 5.30 |  |  |  |
|  |  | Total | 151 | 100.00 |  |  |  |
| Severe patients (oxygen saturation <92%) received blood gas analysis | | |  |  | A+C | 0 | 69.54 |
|  | A | yes | 104 | 68.87 |  |  |  |
|  | B | no | 46 | 30.46 |  |  |  |
|  | C | non-severe patient | 1 | 0.66 |  |  |  |
|  |  | Total | 151 | 100.00 |  |  |  |
| Timeliness of sputum and blood culture | | |  |  | A | 0 | 77.48 |
|  | A | within 24 hours | 117 | 77.48 |  |  |  |
|  | B | greater than 24 hours | 30 | 19.87 |  |  |  |
|  | C | no records | 4 | 2.65 |  |  |  |
|  |  | Total | 151 | 100.00 |  |  |  |
| Timely and appropriate use of antibiotics | | |  |  | A | 1 | 92.00 |
|  | A | appropriate use within 4 hours | 138 | 92.00 |  |  |  |
|  | B | appropriate use after 4 hours | 12 | 8.00 |  |  |  |
|  |  | Total | 150 | 100.00 |  |  |  |
| Appropriate treatment update at 72 hours | | |  |  |  | 0 |  |
|  | A | maintaining original treatment or changing to oral medicine if effective treatment are shown at 72 hours | 139 | 98.68 | A+B |  | 98.01 |
|  | B | taking pathological examination and changing treatment scheme if effective treatment are not shown at 72 hours | 9 | 1.32 |  |  |  |
|  | C | no assessment on treatment effectiveness at 72 hours | 1 | 100.00 |  |  |  |
|  | D | no records | 2 |  |  |  |  |
|  |  | total | 151 | 99.29 |  |  |  |
| Antibiotic treatment is reasonable（7~14days） | | |  |  | A | 0 | 80.79 |
|  | A | reasonable | 122 | 80.79 |  |  |  |
|  | B | not reasonable | 29 | 19.21 |  |  |  |
|  | | Total | 151 | 100.00 |  |  |  |
| Received health education | | |  |  | A | 0 | 98.01 |
|  | A | yes | 148 | 98.01 |  |  |  |
|  | B | no | 3 | 1.99 |  |  |  |
|  |  | Total | 151 | 100.00 |  |  |  |
| Appropriate length of stay | | |  |  | A | 0 | 100.00 |
|  | A | <=7 days | 151 | 100.00 |  |  |  |
|  | B | >7 days | 0 | 0.00 |  |  |  |
|  |  | Total | 151 | 100.00 |  |  |  |
| Overall | |  |  |  | A | 1 | 38.67 |
|  | A | meeting all guideline | 58 | 38.67 |  |  |  |
|  | B | not meeting all guideline | 92 | 61.33 |  |  |  |
|  |  | Total | 150 |  |  |  |  |

† ICD-10: J13、J14、J15、J18.

Table S2 Analysis on Compliance Rate for AMI Inpatient Care (n=97)^†^

| Key process indicators | | | N | Percentage（%） | Rule | Missing Data | Compliance rate (%) |
| --- | --- | --- | --- | --- | --- | --- | --- |
| Timely use of aspirin or clopidogrel in appropriate dosage | | |  |  | A | 2 | 68.42 |
|  | A | using aspirin or clopidogrel just after arrival and with 300 mg | 65 | 68.42 |  |  |  |
|  | B | using aspirin or clopidogrel just after arrival | 2 | 2.11 |  |  |  |
|  | C | using aspirin or clopidogrel with 300 mg | 2 | 2.11 |  |  |  |
|  | D | using aspirin or clopidogrel not with 300mg | 9 | 9.47 |  |  |  |
|  | E | no record | 17 | 17.89 |  |  |  |
|  |  | Total | 95 | 100.00 |  |  |  |
| Evaluation of left ventricular function within 24 hours of admission | | |  |  | A | 1 | 84.38 |
|  | A | yes | 81 | 84.38 |  |  |  |
|  | B | no | 15 | 15.63 |  |  |  |
|  |  | Total | 96 | 100.00 |  |  |  |
| Reassessment of patient condition within one week before discharge | | |  |  | A | 1 | 0 |
|  | A | yes | 0 | 0.00 |  |  |  |
|  | B | no | 96 | 100.00 |  |  |  |
|  |  | Total | 96 | 100.00 |  |  |  |
| Reperfusion therapy | | |  |  | A+B | 8 | 75.28 |
|  | A | reperfusion therapy for STEMI or LBBB patients ^‡^ | 67 | 75.28 |  |  |  |
|  | B | no reperfusion therapy for non STEMI and no LBBB patients | 0 | 0.00 |  |  |  |
|  | C | no reperfusion therapy for STEMI or LBBB patients | 21 | 23.60 |  |  |  |
|  | D | reperfusion therapy for no STEMI and no LBBB patients | 1 | 1.12 |  |  |  |
|  |  | Total | 89 | 100.00 |  |  |  |
| Thrombolytic therapy within 30 minutes of admission | | |  |  | A+C+D | 0 | 5.15 |
|  | A | Implement within 30 minutes of admission | 5 | 5.15 |  |  |  |
|  | B | Implement 30 minutes later of admission | 2 | 2.06 |  |  |  |
|  | C | No thrombolytic therapy because of no indication | 0 | 0.00 |  |  |  |
|  | D | No thrombolytic therapy because of having contraindication | 0 | 0.00 |  |  |  |
|  | E | Improper treatment | 90 | 92.78 |  |  |  |
|  |  | Total | 97 | 100.00 |  |  |  |
| PCI within 90 minutes of admission | | |  |  | A+C+D | 0 | 0.00 |
|  | A | Implement of PCI within 90 minutes of admission | 0 | 0.00 |  |  |  |
|  | B | Implement 90 minutes later of admission | 72 | 74.23 |  |  |  |
|  | C | No PCI because of no indication | 0 | 0.00 |  |  |  |
|  | D | No PCI because of having contraindication | 0 | 0.00 |  |  |  |
|  | E | Improper treatment | 25 | 25.77 |  |  |  |
|  |  | Total | 97 | 100.00 |  |  |  |
|  |  |  |  |  |  |  |  |
| Use of β-blocker within 60 minutes of admission | | |  |  | A | 1 | 25.00 |
|  | A | within 60 minutes | 24 | 25.00 |  |  |  |
|  | B | within 24 hours | 40 | 41.67 |  |  |  |
|  | C | no records | 30 | 31.25 |  |  |  |
|  | D | have contraindications | 2 | 2.08 |  |  |  |
|  |  | Total | 96 | 100.00 |  |  |  |
| Use of aspirin during hospitalization | | |  |  | A | 1 | 93.75 |
|  | A | yes | 90 | 93.75 |  |  |  |
|  | B | no | 6 | 6.25 |  |  |  |
|  |  | Total | 96 | 100.00 |  |  |  |
| Use of β-blocker during hospitalization | | |  |  | A | 0 | 73.20 |
|  | A | yes | 71 | 73.20 |  |  |  |
|  | B | no | 26 | 26.80 |  |  |  |
|  |  | Total | 97 | 100.00 |  |  |  |
| USE of ACEI or ARB during hospitalization ^†^ | | |  |  | A | 0 | 73.20 |
|  | A | yes | 71 | 73.20 |  |  |  |
|  | B | no | 26 | 26.80 |  |  |  |
|  | | Total | 97 | 100.00 |  |  |  |
| Use of statins during hospitalization | | |  |  |  | 0 | 92.78 |
|  | A | yes | 90 | 92.78 | A |  |  |
|  | B | no | 7 | 7.22 |  |  |  |
|  |  | Total | 97 | 100.00 |  |  |  |
| Cholesterol test and lipid lowering therapy | | |  |  | A+B | 0 | 26.80 |
|  | A | Cholesterol test with high LDL (>3.6mmol/L) and lipid lowering therapy | 21 | 21.65 |  |  |  |
|  | B | Cholesterol test without high LDL and no lipid lowering therapy | 5 | 5.15 |  |  |  |
|  | C | Cholesterol test with high LDL and no lipid lowering therapy | 0 | 0.00 |  |  |  |
|  | D | lipid lowering therapy with no cholesterol test or low LDL | 71 | 73.20 |  |  |  |
|  |  | Total | 97 | 100.00 |  |  |  |
| Advised to continue to use aspirin after discharge | | |  |  | A | 0 | 80.41 |
|  | A | yes | 78 | 80.41 |  |  |  |
|  | B | no | 19 | 19.59 |  |  |  |
|  |  | Total | 97 | 100.00 |  |  |  |
| Advised to continue to use β-blocker after discharge | | |  |  | A | 1 | 61.46 |
|  | A | yes | 59 | 61.46 |  |  |  |
|  | B | no | 37 | 38.54 |  |  |  |
|  |  | Total | 96 | 100.00 |  |  |  |
| Advised to continue to use ACEI or ARB after discharge ^#^ | | |  |  | A | 3 | 64.89 |
|  | A | yes | 61 | 64.89 |  |  |  |
|  | B | no | 33 | 35.11 |  |  |  |
|  |  | Total | 94 | 100.00 |  |  |  |
| Advised to continue to use statin after discharge | | |  |  | A | 0 | 78.35 |
|  | A | yes | 76 | 78.35 |  |  |  |
|  | B | no | 21 | 21.65 |  |  |  |
|  |  | Total | 97 | 100.00 |  |  |  |
|  |  |  |  |  |  |  |  |
| Advised to no smoking, having exercise, healthy eating, weight  control, proper treatment of recurrence or worsening, etc | | | | | A | 82.00 | 84.54 |
|  | A | yes | 82 | 84.54 |  |  |  |
|  | B | no | 15 | 15.46 |  |  |  |
|  |  | Total | 97 | 100.00 |  |  |  |
| Smoking cessation counseling | | |  |  | A | 0 | 84.54 |
|  | A | yes | 82 | 84.54 |  |  |  |
|  | B | no | 15 | 15.46 |  |  |  |
|  |  | Total | 97 | 100.00 |  |  |  |
| Provided with writen instruction on secondary prevention in discharge summary | | |  |  | A | 0 | 83.51 |
|  | A | yes | 81 | 83.51 |  |  |  |
|  | B | no | 16 | 16.49 |  |  |  |
|  |  | Total | 97 | 100.00 |  |  |  |
| Overall | |  |  |  | A | 17 | 0.00 |
|  | A | meeting all guideline | 0 | 0.00 |  |  |  |
|  | B | not meeting all guideline | 80 | 100.00 |  |  |  |
|  |  | Total | 80 | 100.00 |  |  |  |

† ICD-10: I21.0I21.1I21.2I21.3I21.9;

‡ STEMI: ST elevation myocardial infarction; LBBB: left bundle branch block;

# ACEI:Angiotensin converting enzyme inhibitors，ARB：Angiotensin II receptor blockers.

Table S3 Analysis on Compliance Rate for Heart Failure Inpatient Care (n=145)^†^

| Key process indicators | | | N | Percentage（%） | Rule | Missing Data | Compliance rate (%) |
| --- | --- | --- | --- | --- | --- | --- | --- |
| Assessment of left ventricular function within 24 hours of admission | | |  |  | A | 12 | 77.44 |
|  | A | yes | 103 | 77.44 |  |  |  |
|  | B | no | 30 | 22.56 |  |  |  |
|  |  | Total | 133 | 100.00 |  |  |  |
| Assessment of left ventricular function one week prior to discharge | | |  |  | A | 24 | 0.83 |
|  | A | yes | 1 | 0.83 |  |  |  |
|  | B | no | 120 | 99.17 |  |  |  |
|  |  | Total | 121 | 100.00 |  |  |  |
| Timely use of diuretics and potassium agents | | |  |  | A+B+D | 5 | 96.43 |
|  | A | use of diuretics and potassium agents immediately after arrival | 135 | 96.43 |  |  |  |
|  | B | use of diuretics and potassium agents within 60 minutes | 0 | 0.00 |  |  |  |
|  | C | no use of diuretics and potassium agents within 60 minutes | 5 | 3.57 |  |  |  |
|  | D | having contraindications | 0 | 0.00 |  |  |  |
|  |  | Total | 140 | 100.00 |  |  |  |
| Timely use of ACEI or ARB ^‡^ | | |  |  | A+B+D | 0 | 86.90 |
|  | A | use of ACEI or ARB immediately after admission | 126 | 86.90 |  |  |  |
|  | B | use of ACEI or ARB within 60 minutes of admission | 0 | 0.00 |  |  |  |
|  | C | no use of ACEI or ARB within 60 minutes | 19 | 13.10 |  |  |  |
|  | D | having contraindications | 0 | 0.00 |  |  |  |
|  |  | Total | 145 | 100.00 |  |  |  |
| Use of β-blockers only for patients with CHF ^#^ | | |  |  | A+D | 12 | 19.55 |
|  | A | use of β-blockers for patients with CHF | 25 | 18.80 |  |  |  |
|  | B | use of β-blockers for patients with AHF | 0 | 0.00 |  |  |  |
|  | C | use of β-blockers for other patients | 43 | 32.33 |  |  |  |
|  | D | no use of β-blockers for patients without CHF or with AHF | 1 | 0.75 |  |  |  |
|  | E | no use of β-blocker for patients with CHF but without AHF | 64 | 48.12 |  |  |  |
|  |  | Total | 133 | 100.00 |  |  |  |
| Use of aldosterone receptor blockers only for patients with severe health failure^※^ | | |  |  | A+C | 3 | 85.21 |
|  | A | use of aldosterone receptor blockers for patients with severe health failure | 121 | 85.21 |  |  |  |
|  | B | no use of aldosterone receptor blockers for patients with severe health failure | 21 | 14.79 |  |  |  |
|  | C | non severe health failure | 0 | 0.00 |  |  |  |
|  |  | Total | 142 | 100.00 |  |  |  |
| Continued use of diuretics during hospitalization | | |  |  | A | 0 | 93.10 |
|  | A | yes | 135 | 93.10 |  |  |  |
|  | B | no | 10 | 6.90 |  |  |  |
|  |  | Total | 145 | 100.00 |  |  |  |
|  |  |  |  |  |  |  |  |
|  |  |  |  |  |  |  |  |
|  |  |  |  |  |  |  |  |
| Continued use of ACEI or ARB during hospitalization | | |  |  | A | 1 | 86.81 |
|  | A | yes | 125 | 86.81 |  |  |  |
|  | B | no | 19 | 13.19 |  |  |  |
|  |  | Total | 144 | 100.00 |  |  |  |
| Continued use of β-blocker during hospitalization | | |  |  | A+C | 16 | 53.49 |
|  | A | yes | 69 | 53.49 |  |  |  |
|  | B | no | 58 | 44.96 |  |  |  |
|  | C | contraindication | 2 | 1.55 |  |  |  |
|  |  | Total | 129 | 100.00 |  |  |  |
| Continued use of aldosterone receptor blockers during hospitalization | | |  |  | A | 6 | 84.17 |
|  | A | yes | 117 | 84.17 |  |  |  |
|  | B | no | 22 | 15.83 |  |  |  |
|  |  | Total | 139 | 100.00 |  |  |  |
| Advised to use diuretics after discharge | | |  |  | A | 6 | 82.01 |
|  | A | yes | 114 | 82.01 |  |  |  |
|  | B | no | 25 | 17.99 |  |  |  |
|  |  | Total | 139 | 100.00 |  |  |  |
| Advised to use ACEI or ARB after discharge | | |  |  | A | 5 | 75.71 |
|  | A | yes | 106 | 75.71 |  |  |  |
|  | B | no | 34 | 24.29 |  |  |  |
|  |  | Total | 140 | 100.00 |  |  |  |
| Advised to use β-blocker after discharge | | |  |  | A | 19 | 53.97 |
|  | A | yes | 68 | 53.97 |  |  |  |
|  | B | no | 58 | 46.03 |  |  |  |
|  |  | Total | 126 | 100.00 |  |  |  |
| Advised to use aldosterone receptor blockers after discharge | | |  |  | A | 11 | 77.61 |
|  | A | yes | 104 | 77.61 |  |  |  |
|  | B | no | 30 | 22.39 |  |  |  |
|  |  | Total | 134 | 100.00 |  |  |  |
| Record of heart failure education | | |  |  | A | 0 | 100.00 |
|  |  | yes | 145 | 100.00 |  |  |  |
|  |  | no | 0 | 0.00 |  |  |  |
|  |  | Total | 145 | 100.00 |  |  |  |
| Assessment of cardiac function and living ability, and guidance activities after admission | | |  |  | A | 0 | 100.00 |
|  | A | yes | 145 | 100.00 |  |  |  |
|  | B | no | 0 | 0.00 |  |  |  |
|  |  | Total | 145 | 100.00 |  |  |  |
| Proper observation of patients (including symptoms, vital signs, water balance, weight, edema), provision of laboratory tests, and advice on diet and body-position after admission. | | |  |  | A | 1 | 99.31 |
|  | A | yes | 144 | 99.31 |  |  |  |
|  | B | no | 0 | 0.00 |  |  |  |
|  |  | Total | 144 | 99.31 |  |  |  |
|  |  |  |  |  |  |  |  |
|  |  |  |  |  |  |  |  |
|  |  |  |  |  |  |  |  |
| Assessment of tobacco and alcohol addiction after admission and Patient advised to quit smoking and to restrict alcohol consumption | | |  |  | A | 0 | 54.48 |
|  | A | yes | 79 | 54.48 |  |  |  |
|  | B | no | 66 | 45.52 |  |  |  |
|  |  | Total | 145 | 100.00 |  |  |  |
| Patient received psychological counseling | | |  |  | A | 0 | 58.62 |
|  |  | yes | 85 | 58.62 |  |  |  |
|  |  | no | 60 | 41.38 |  |  |  |
|  |  | Total | 145 | 100.00 |  |  |  |
| Patient advised on activity limitations after discharge | | |  |  | A | 1 | 100.00 |
|  |  | yes | 144 | 100.00 |  |  |  |
|  |  | no | 0 | 0.00 |  |  |  |
|  |  | Total | 144 | 100.00 |  |  |  |
| Patient received dietary and body-position guidance prior to discharge | | |  |  | A | 2 | 100.00 |
|  |  | yes | 143 | 100.00 |  |  |  |
|  |  | no | 0 | 0.00 |  |  |  |
|  |  | Total | 143 | 100.00 |  |  |  |
| Patient advised to quit smoking and to restrict alcohol consumption prior to discharge | | |  |  | A | 2 | 98.60 |
|  |  | yes | 141 | 98.60 |  |  |  |
|  |  | no | 2 | 1.40 |  |  |  |
|  |  | Total | 143 | 100.00 |  |  |  |
| Overall | |  |  |  | A | 69 | 0.00 |
|  | A | meeting all guideline | 0 | 0.00 |  |  |  |
|  | B | not meeting all guideline | 76 | 100.00 |  |  |  |
|  |  | Total | 76 | 100.00 |  |  |  |

† ICD-10:I11,I12,I13;

‡ ACEI:Angiotensin converting enzyme inhibitor, ARB：Angiotensin II receptor blockers Severe heart failure refers to the New York Heart Association (NYHA) cardiac function proposed test (NYHA functional) III, IV level of the patients;

# CHF: Chronic heart failure, AHF: Acute heart failure;

※ Severe heart failure refers to the New York Heart Association (NYHA) cardiac function proposed test (NYHA functional) III, IV level of the patients;

Table S4 Analysis on Compliance Rate for Caesarean Inpatient Care (n=146) ^†^

| Key process indicators | | | N | Percentage（%） | Rule | Missing Data | Compliance rate (%) |
| --- | --- | --- | --- | --- | --- | --- | --- |
| Appropriate indication for planned C-section | | |  |  | A~G | 0 | 100.00 |
|  | A | Demand from pregnant women or their relatives | 62 | 42.47 |  |  |  |
|  | B | Oligohydramnios | 16 | 10.96 |  |  |  |
|  | C | Fetal factors | 15 | 10.27 |  |  |  |
|  | D | Chronic fetal distress | 15 | 10.27 |  |  |  |
|  | E | Cephalopelvic disproportion | 12 | 8.22 |  |  |  |
|  | F | Complications that affect vaginal delivery | 10 | 6.85 |  |  |  |
|  | G | Other appropriate indications | 16 | 10.96 |  |  |  |
|  | H | no appropriate indication | 0 | 0.00 |  |  |  |
|  |  | Total | 146 | 100.00 |  |  |  |
| Preoperative examination completed within 2 days | | |  |  | A | 0 | 99.32 |
|  | A | yes | 145 | 99.32 |  |  |  |
|  | B | no | 1 | 0.68 |  |  |  |
|  |  | Total | 146 | 100.00 |  |  |  |
| Prophylactic use of first generation cephalosporin antibiotics | | |  |  | A | 0 | 62.33 |
|  | A | yes | 91 | 62.33 |  |  |  |
|  | B | no | 55 | 37.67 |  |  |  |
|  |  | Total | 146 | 100.00 |  |  |  |
| Withdraw of prophylactic antibiotics within 72 hours after delivery | | |  |  | A | 0 | 86.30 |
|  | A | yes | 126 | 86.30 |  |  |  |
|  | B | no | 20 | 13.70 |  |  |  |
|  |  | Total | 146 | 100.00 |  |  |  |
| The timeliness of operation time | | |  |  | A | 0 | 61.64 |
|  | A | ≤2day | 90 | 61.64 |  |  |  |
|  | B | >2day | 56 | 38.36 |  |  |  |
|  |  | Total | 146 | 100.00 |  |  |  |
| Delivery within 2 days of admission | | |  |  | A | 0 | 82.88 |
|  | A | ≤2days | 91 | 62.33 |  |  |  |
|  | B | >2days with reasons | 30 | 20.55 |  |  |  |
|  | C | >2days without reasons | 25 | 17.12 |  |  |  |
|  |  | Total | 146 | 100.00 |  |  |  |
| Appropriate anesthesia | | |  |  | A | 1 | 65.52 |
|  | A | Epidural or combined spinal epidural | 95 | 65.52 |  |  |  |
|  | B | Spinal anesthesia | 50 | 34.48 |  |  |  |
|  |  | Total | 145 | 100.00 |  |  |  |
| Appropriate use of oxytocin during procedure | | |  |  | A | 0 | 80.82 |
|  | A | 10ug or 20ug | 118 | 80.82 |  |  |  |
|  | B | 30ug or 40ug | 19 | 13.01 |  |  |  |
|  | C | >40ug | 8 | 5.48 |  |  |  |
|  | D | no use | 1 | 0.68 |  |  |  |
|  |  | Total | 146 | 100.00 |  |  |  |
| Post-operative length of stay | | |  |  | A | 3 | 99.30 |
|  | A | ≤7day | 142 | 99.30 |  |  |  |
|  | B | >7day | 1 | 0.70 |  |  |  |
|  |  | Total | 143 | 100.00 |  |  |  |
|  |  |  |  |  |  |  |  |
| In accordance with discharge standard | | |  |  | A | 4 | 100.00 |
|  | A | yes | 142 | 100.00 |  |  |  |
|  | B | no | 0 | 0.00 |  |  |  |
|  |  | Total | 142 | 100.00 |  |  |  |
| Patient received health education prior to discharge | | |  |  | g | 2 | 88.89 |
|  | A | yes | 128 | 88.89 |  |  |  |
|  | B | no | 16 | 11.11 |  |  |  |
|  |  | Total | 144 | 100.00 |  |  |  |
| Overall | |  |  |  | A | 2 | 22.22 |
|  | A | meeting all guideline | 32 | 22.22 |  |  |  |
|  | B | not meeting all guideline | 112 | 77.78 |  |  |  |
|  |  | Total | 144 | 100.00 |  |  |  |

† ICD-10:the first diagnosis of underlying section of uterus caesarian operation, ICD-9-CM-3：74.1Surgical coders.

Table S5 Analysis on Compliance Rate for Type-2 Diabetic Inpatient Care (n=137)^†^

| Key process indicators | | | No | Percentage（%） | Rule | Missing Data | Compliance rate (%) |
| --- | --- | --- | --- | --- | --- | --- | --- |
| Routine examination within 24 hours after admission | | |  |  | A | 0 | 100.00 |
|  | A | yes | 137 | 100.00 |  |  |  |
|  | B | no | 0 | 0.00 |  |  |  |
|  |  | Total | 137 | 100.00 |  |  |  |
| Blood glucose monitoring 7 times per day | | |  |  | A+B | 0 | 63.50 |
|  | A | monitoring 7 times per day | 70 | 51.09 |  |  |  |
|  | B | dynamic monitoring of blood glucose | 17 | 12.41 |  |  |  |
|  | C | monitoring 4 times per day | 46 | 33.58 |  |  |  |
|  | D | no monitoring | 4 | 2.92 |  |  |  |
|  |  | Total | 137 | 100.00 |  |  |  |
| HbA1c test | | |  |  | A | 0 | 91.24 |
|  | A | yes | 125 | 91.24 |  |  |  |
|  | B | no | 12 | 8.76 |  |  |  |
|  |  | Total | 137 | 100.00 |  |  |  |
| Glycosylated Serum Protein（Fructosamine）test | | |  |  | A | 1 | 55.15 |
|  | A | yes | 75 | 55.15 |  |  |  |
|  | B | no | 61 | 44.85 |  |  |  |
|  |  | Total | 136 | 100.00 |  |  |  |
| OGTT and insulin or C peptide release test^‡^ | | |  |  | A+B | 0 | 58.39 |
|  | A | accord with the standard test(5 points) | 17 | 12.41 |  |  |  |
|  | B | only test fast and 2-hr blood glucose | 63 | 45.99 |  |  |  |
|  | C | Arginine release test | 17 | 12.41 |  |  |  |
|  | D | other type of tests | 27 | 19.71 |  |  |  |
|  | E | no records | 13 | 9.49 |  |  |  |
|  |  | Total | 137 | 100.00 |  |  |  |
| Eye fundus examination | | |  |  | A | 0 | 62.04 |
|  | A | yes | 85 | 62.04 |  |  |  |
|  | B | no | 52 | 37.96 |  |  |  |
|  |  | Total | 137 | 100.00 |  |  |  |
| Nerve system examination | | |  |  | A | 0 | 39.42 |
|  | A | yes | 54 | 39.42 |  |  |  |
|  | B | no | 83 | 60.58 |  |  |  |
|  |  | Total | 137 | 100.00 |  |  |  |
| Renal function examination | | |  |  | A | 0 | 83.21 |
|  | A | yes | 114 | 83.21 |  |  |  |
|  | B | no | 23 | 16.79 |  |  |  |
|  |  | Total | 137 | 100.00 |  |  |  |
| Heart ultrasound examination | | |  |  | A | 0 | 71.53 |
|  | A | yes | 98 | 71.53 |  |  |  |
|  | B | no | 39 | 28.47 |  |  |  |
|  |  | Total | 137 | 100.00 |  |  |  |
| Carotid artery and lower extremity vascular ultrasound examination | | |  |  | A | 0 | 72.99 |
|  | A | yes | 100 | 72.99 |  |  |  |
|  | B | no | 37 | 27.01 |  |  |  |
|  | | Total | 137 | 100.00 |  |  |  |
|  | |  |  |  |  |  |  |
|  | |  |  |  |  |  |  |
|  | |  |  |  |  |  |  |
| Blood glucose test analyzed | | |  |  | A | 0 | 97.08 |
|  | A | yes | 133 | 97.08 |  |  |  |
|  | B | no | 4 | 2.92 |  |  |  |
|  |  | Total | 137 | 100.00 |  |  |  |
| Evaluation at 72 hours after hypoglycemic treatment | | |  |  | A | 0 | 85.40 |
|  | A | yes | 117 | 85.40 |  |  |  |
|  | B | no | 20 | 14.60 |  |  |  |
|  |  | Total | 137 | 100.00 |  |  |  |
| Record of drug selection reasons | | |  |  | A | 0 | 99.27 |
|  | A | yes | 136 | 99.27 |  |  |  |
|  | B | no | 1 | 0.73 |  |  |  |
|  |  | Total | 137 | 100.00 |  |  |  |
| Record of secondary prevention and health education provided to patient | | |  |  | A | 0 | 98.54 |
|  | A | yes | 135 | 98.54 |  |  |  |
|  | B | no | 2 | 1.46 |  |  |  |
|  |  | Total | 137 | 100.00 |  |  |  |
| In accordance with discharge standard | | |  |  | A | 0 | 96.35 |
|  | A | yes | 132 | 96.35 |  |  |  |
|  | B | no | 5 | 3.65 |  |  |  |
|  |  | Total | 137 | 100.00 |  |  |  |
| Appropriate length of stay | | |  |  | A+B | 0 | 88.32 |
|  | A | ≤14day | 93 | 67.88 |  |  |  |
|  | B | >14day with reasons | 28 | 20.44 |  |  |  |
|  | C | >14day without reasons | 16 | 11.68 |  |  |  |
|  |  | Total | 137 | 100.00 |  |  |  |
| Overall | |  |  |  |  |  |  |
|  | A | meeting all guideline | 3 | 2.21 | A | 1 | 2.21 |
|  | B | not meeting all guideline | 133 | 97.79 |  |  |  |
|  |  | Total | 136 | 100.00 |  |  |  |

† ICD-10: E11.2- E11.9;

‡ OGTT：Oral glucose tolerance test.
